# A Novel Nutraceutical Formulation Can Improve Motor Activity and Decrease the Stress Level in a Murine Model of Middle-Age Animals

**DOI:** 10.3390/jcm10040624

**Published:** 2021-02-06

**Authors:** Dimitris Tsoukalas, Ovidiu Zlatian, Mihaela Mitroi, Elisavet Renieri, Aristidis Tsatsakis, Boris Nikolaevich Izotov, Florin Burada, Simona Sosoi, Emilia Burada, Ana Maria Buga, Ion Rogoveanu, Anca Oana Docea, Daniela Calina

**Affiliations:** 1 Department of Clinical Pharmacy, University of Medicine and Pharmacy of Craiova, 200349 Craiova, Romania; 2 Metabolomic Medicine, Health Clinic for Autoimmune and Chronic Diseases, 10674 Athens, Greece; 3 European Institute of Nutritional Medicine (E.I.Nu.M.), 00198 Rome, Italy; 4 Department of Microbiology, University of Medicine and Pharmacy of Craiova, 200349 Craiova, Romania; ovidiu.zlatian@gmail.com; 5 ENT Department, University of Medicine and Pharmacy of Craiova, 200349 Craiova, Romania; mihaela.mitroi48@gmail.com; 6 Laboratory of Toxicology and Forensic Sciences, Medical School, University of Crete, 71003 Heraklion, Greece; elisavet_renieri@hotmail.com (E.R.); aristsatsakis@gmail.com (A.T.); 7 Department of Analytical and Forensic Medical Toxicology, Sechenov University, 119991 Moscow, Russia; bn38@mail.ru; 8 Human Genomics Laboratory, University of Medicine and Pharmacy of Craiova, 200349 Craiova, Romania; buradaflorin@gmail.com (F.B.); simona.sosoi@umfcv.ro (S.S.); 9 Department of Physiology, University of Medicine and Pharmacy of Craiova, 200349 Craiova, Romania; emilia.burada@umfcv.ro; 10 Department of Biochemistry, University of Medicine and Pharmacy of Craiova, 200349 Craiova, Romania; anamaria.buga@umfcv.ro; 11 Department of Internal Medicine, University of Medicine and Pharmacy of Craiova, 200349 Craiova, Romania; ionirogoveanu@gmail.com; 12 Department of Toxicology, University of Medicine and Pharmacy of Craiova, 200349 Craiova, Romania

**Keywords:** *Centella asiatica* L., vitamin C, zinc, vitamin D, anxiety levels, motor performance, ageing

## Abstract

Ageing is a genetically programmed physiological process that is modulated by numerous environmental factors, associated with decreasing physiological function, decreasing reproductive rate and increasing age-related mortality rate. Maintaining mobility performance and physical function in the elderly is the main objective of the successful ageing concept. In this study, we aimed to evaluate the beneficial effect of a novel nutraceutical formulation containing *Centella asiatica* L. extract, vitamin C, zinc and vitamin D3 (as cholecalciferol) on motor activity and anxiety with the use of a murine model of old animals, as a means of providing proof for clinical use in the elderly, for enhancing physical strength and improving life quality. Eighteen Sprague Dawley 18 months old male rats were divided into three groups and received corn oil (the control group) or 1 capsule/kg bw Reverse supplement (treatment group 1) or 2 capsules/kg bw Reverse supplement (treatment group 2), for a period of 3 months. The Reverse supplement (Natural Doctor S.A, Athens, Greece) contains 9 mg *Centella asiatica* L. extract, vitamin C (200 mg as magnesium ascorbate), zinc (5 mg as zinc citrate), vitamin D3 (50 µg as cholecalciferol) per capsule. Before and after the treatment, the motor function and behavioral changes for anxiety and depression were evaluated using the open-field test, elevated plus-maze test and rotarod test. The supplementation with Reverse (Natural Doctor S.A) supplement can improve the locomotor activity in old rats in a dose-dependent manner, as demonstrated by an increase in the latency to leave from the middle square, in the number of rearings in the open field test, in the time spent in the open arms and time spent in the center in the elevated plus-maze test and the latency to all in all three consecutive trials in the rotarod test. Stress also decreased significantly in a dose-dependent manner, following the treatment with Reverse supplement, as was demonstrated by the decrease in the number of groomings at the open field test and time spent in the dark and the number of groomings at the elevated plus-maze test.

## 1. Introduction

Ageing is a genetically programmed physiological process that is modulated by a variety of environmental factors and is associated with decreasing physiological function, decreasing reproductive rate and increasing mortality rate [1]. With the increase of lifespan in the last years, the research in gerontology has mainly focused on improving the life quality of the elderly and a new concept known as “successful ageing,” first introduced in 1961 by Robert J. Havighurst, gathered attention [2]. The activity theory as described by the successful ageing concept depicts the need for prolonging the middle age activities and attitude for as long as possible [2]. In this context, it is crucial to prevent the decline of physical and cognitive functions that start from middle age, along with maintaining engagement with life, while the need for preventing disease and disability in the elderly becomes imperative [3].

State of the art in ageing and gerontology focuses on defining the metrics for assessing successful ageing and several outcomes have been validated for the evaluation of health and functional abilities improvement [4]. Deterioration in mobility performance and physical function are considered the main indicators of functional decline, health impairment and mortality in the elderly [5,6].

Several interventions have been previously studied for preventing cognitive and physical decline and maintaining physical performance, mobility and independence in the elderly such as a healthy lifestyle [7,8], meditation [9,10,11], cognitive training [12], pharmaceuticals [13], plant extracts [14,15,16,17] and nutraceuticals [14,18,19].

Antioxidants consumption has been associated with preventing mobility and physical decline especially in the elderly [20]. Vitamin C is a water-soluble vitamin with potent antioxidant properties. Several studies in the elderly showed that vitamin C is implicated in maintaining human bone homeostasis through decreasing oxidative stress [21]. Vitamin D is a fat-soluble vitamin found in certain foods and is also produced endogenously at skin level under ultraviolet rays action. In modern society, vitamin D deficiency is reported in the population of all ages. It has complex effects on the human organism, from influencing bone metabolism by increasing calcium absorption to modulating gene expression, decreasing inflammation, as well as mitigating the risk of infection and cancer and increasing immunity [22]. Additionally, a normal level of vitamin D in the elderly is associated with decreased fracture risk and improved physical activity [23]. Zinc is a trace mineral that acts as a cofactor for many metalloenzymes. It is also implicated in membranes’ stability and several regulatory and catalytic functions in the human body [24]. It plays an important role in bone formation and remodeling and decreased zinc level is associated with a decrease in bone formation due to inhibition of collagen, alkaline phosphatase and osteoblastic activity [25]. *Centella asiatica* (L.) urban is a plant of the Apiaceae family found mainly in Asiatic countries such as China, India and Thailand and is known for its wide variety of beneficial health effects, from alleviating asthma, eczemas [26], ulcers [27], headache, skin wounds [28] to improving cognitive function [29] and diminishing anxiety [30] and depression. It holds a high antioxidant potential [31] and is associated with beneficial effects on physical strength and increased life quality.

In this study, we aimed to evaluate for the first time the beneficial effect of a new nutraceutical formulation, Reverse, containing *Centella asiatica* L. extract, vitamin C, zinc and vitamin D3 (as cholecalciferol), on motor activity and anxiety, with the use of a murine model of middle-age animals. The study intends to build evidence for clinical use in middle-age adults, as a means of enhancing physical strength and overall improving quality of life using the dose equivalent in animals for the current doses approved for human use as food supplement.

## 2. Materials and Methods

### 2.1. Animal Study Design

Eighteen Sprague Dawley male rats, 18 months old with a body weight ranging between 500 and 580 g were randomly divided into 3 groups, with 6 rats per group. Male rats were selected as they are extensively used in animal experiments and the variability is reduced compared to female animals that go through an estrous cycle which is a faster version of the human menstrual cycle that determine the change in hormone concentration in a four-to five day schedule, so if the female rats are at different points in their estrous cycle, their response can vary too much [32]. The animals were obtained from the University of Medicine and Pharmacy of Craiova Animal House, Craiova Romania, authorization number 76/20.04.2016. The protocol of the animal experiment was approved by the Ethical Committee of the University of Medicine and Pharmacy of Craiova, Craiova, Romania, number 102/23.09.2019 and all the procedures were in accordance with the European directives for the animal experiments (EU Directive 2010/63/EU as amended by Regulation EU 2019/1010). All animals were acclimatized to the new housing conditions for two weeks prior to the experiment. The animals were kept in standard conditions with controlled temperature and humidity during the experiment, with 12 h dark/light cycle and received free access to standard animal feed and tap water.

The animals received as an intervention for 3 months the following treatment:

- The treatment group 1 (6 rats per group)—received 1 capsule/kg bw of Reverse (Natural Doctor S.A., Athens, Greece) supplement approved by the Greek National Organization for Medicines—Registration No: 6704/21-1-2020.
- The treatment group 2 (6 rats per group)—received 2 capsule/kg bw of Reverse (Natural Doctor S.A., Athens, Greece) supplement per day for 3 months.
- The control group (6 rats per group)—received every day 1.5 mL corn oil once per day for 3 months.

### 2.2. Treatment Dose Selection and Administration

The Reverse (Natural Doctor S.A.) supplement is approved by the Greek National Organization for Medicines—Registration No: 6704/21-1-2020 as a food supplement in doses of 1 or 2 capsules/day for adults. The supplement contains 9 mg *Centella asiatica* L. extract [consisting of a >90% high purity single chemical entity as assessed by HPLC (High Performance Liquid Chromatography) and GC (Gas Chromatography), vitamin C (200 mg as magnesium ascorbate), zinc (5 mg as zinc citrate), vitamin D3 (50 µg as cholecalciferol) per capsule. Cholecalciferol was selected over ergocalciferol since ergocalciferol is less stable and less potent than cholecalciferol. It has been suggested that cholecalciferol is the only vitamin D form that should be given for supplementation [33].

Started from the human doses, we extrapolated the doses that we can test in rats using the correction factor (km) for the used species and the safety factor value for humans. According to the Food and Drug administration guidelines, the correction factor is estimated by dividing the mean body weight (kg) of the used species to the species body surface area (m^2^) and for rats is 6.2. The safety factor value for convert rat doses to humans is 10 [34].

For treatment group 1 that corresponds to the human dose of 1 capsule/day we have the following calculations:

The reference bodyweight for humans is 60 [34], which means the dose for humans = 1/60 capsule per kg bw. The dose in rats = 1/60 × 6.2 × 10 = 1.03 capsule per kg bw that will approximate with 1 capsule per kg bw rat.

For treatment group 2 that corresponds to the human dose of 2 capsules/day we have the following calculations: the dose for humans = 2/60 capsule per kg bw. The dose in rats = 2/60 × 6.2 × 10 = 2.06 capsule per kg bw that will approximate with 2 capsules per kg bw rat.

For the administration to animals, the capsules’ content was suspended in corn oil, used as an inert suspension agent for non-water soluble drugs in animal experiments [35] as a stock suspension with the concentration of 1 capsule/1 mL corn oil or 2 capsules/1 mL corn oil. Each rat received by gavage the equivalent of 1 capsule/kg bw or 2 capsules/kg bw, once per day at the same hour for 3 months as follows. The volume from the stock solution requested according to the equivalent dose for each animal based on the bodyweight was diluted with corn oil till the final volume of 1.5 mL and administered by gavage.

The dose administered was within clinical recommendations relative to body weight and similar to other studies for vitamin C [36,37], vitamin D3 [38,39] and Zinc [40]. For Vitamin D3, the administered dose per capsule are below the Upper Limit, which are the limits that can be safely given without medical supervision. With respect to the safety levels of the ingested dose for vitamin, D the most common concern is the risk of hypercalcemia which might be evoked in cases where serum 25-hydroxyvitamin D levels exceed 700 ng/mL, which is more than seven times higher than the levels of sufficiency [41]. For *Centella asiatica*, there is no established clinical recommendation and the dose used in the current study was much lower than previous studies [42].

### 2.3. Motor Function and Behavioral Evaluation

Before the start of the experiment (baseline) and after 3 months of supplement intervention, the motor function and behavioral changes for anxiety and depression were evaluated using the open-field test, elevated plus-maze test and rotarod test.

The open-field test is widely used in evaluating the locomotor activity and emotional state of rats and mice. The protocol of this test is previously described in Tsatsakis et al. [43]. Briefly, a square arena (100 cm per 100 cm) divided into 25 equal squares was used to evaluate the behavior of the rats in a new environment. The center region of 9 squares was defined as the “internal region,” while the other region of 16 squares was defined as the “external region.” Each animal was placed in the middle square of the arena and its behavior was registered for 5 min with a video camera. The following parameters were evaluated: the locomotor activity (number of rearings that evaluate the spatial orientation activity, number of crossing over squares in internal and external regions and latency to leave from the middle square) and the level of stress (number of grooming acts and number of boluses) [43]. The evaluation was made in blind by two experts and when there were differences between them a new evaluation was done to get to a consensus.

The elevated plus-maze test is a widely used instrument for evaluating the exploratory activity and anxiety level in rats and mice [44]. The equipment is formed from a platform located 50 cm above the floor in a cross shape with 2 open arms and 2 dark close arms with a length of 90 cm from the center. The animal is placed in the center and a video camera is recording its behavior for 5 min. The following parameters were evaluated: the exploratory activity (number of rearings, number of bendings over the edge that evaluate the spatial orientation activity and time spend in the center and open arms) and the level of stress (number of grooming acts and time spend in the dark space of the apparatus) [44]. The evaluation was made in blind by two experts and when there were differences between them a new evaluation was done in order to get to a consensus.

The rotarod test using an accelerating rotarod is used to evaluate the motor performance of rats. The protocol of the test is described in detail in another study [43]. Briefly, we used an apparatus with a 30 cm long and 6 cm diameter rod at 27 cm above the landing table that is attached to a motor and rotated with two speeds, one with a constant speed of 5 rpm and one with an increasing speed from 5 to 40 rpm in 300 s. Each animal was first trained to walk on the rotating rod at 5 rpm for 60 s in 3 consecutive trials separated by 10 min between trials. After training, each animal underwent the 3 consecutive testing procedures, separated by 5 min interval, that evaluates the time spend by each animal on the rod on the accelerated speed. The test was finalized when the animal fell from the rod or clung to the rod and completed a full passive rotation. The latency to fall from the rotating rod in seconds was registered.

### 2.4. Statistical Method

Microsoft Excel (Microsoft Corporation, Redmond, WA 98052, USA) was used to calculate descriptive statistics (mean and standard deviation notation for numeric variables) and the production of the graphs. Further statistical analysis was performed in STATA (StataCorp, College Station, Texas, USA). For the difference of neurological parameters between the control and treatment groups for normally distributed data, we used Analysis of variance (ANOVA) with Dunnett’s adjustment for post-hoc pairwise comparison of means. For data with non-normal distribution, we used the non-parametric equivalents, namely Kruskal Wallis test for group mean difference with Mann-Whitney tests for post-hoc comparisons with Holmes-Sidak adjustment using the Dunn method of adjustment.

In order to evaluate the effect of the two treatments, we performed for each parameter a regression using the difference compared with the baseline values as the dependent variable and the group as an ordinal regressor (the group was coded as 0 for control rats, 1 for rats treated with Treatment 1 and 2 for rats treated with treatment 2). The regression coefficients for the two levels of the group variable estimate the effects of the two treatments compared with the control group. The difference between the treatment groups and the control group is likely due to the effect of the mixture, proving the causality of mixture use that produced the observed change in parameters (that were entered in the regression equation as differences compared to baseline).

We then used a modified form of multiple regression, namely treatment effects analysis, that is intended to prove the causality by comparing the treatment effect at the individual level and averaging the effects. We used the treatment effects among the treated result and the inverse-probability-weighted regression adjustment with a linear model for the outcome and a nonlinear logistic model for treatment modeling. The procedure involves using the regression of the outcome among the controls to estimate the values of the treated subjects if they would not have been treated and use these new generated controls to estimate the treatment effects. The mean value of the outcome in the newly generated controls is called potential outcomes mean and is reported together with the average treatment effect. The potential outcome calculated refers to the probable value in the treated rat if it would not have been treated. It needs to be mentioned that the counterfactual outcome, even though it can be measured after the rat’s treatment but it cannot be measured as if the same rat would not have been treated and it can only be estimated. The difference between these two values is the treatment effect. The procedure accounts for the randomness of outcome effects on an individual and provides better estimates and narrower confidence intervals. All statistical tests used were two-sided and a significant level of 95% (*p* < 0.05).

## 3. Results

### 3.1. The Open Field Test

#### 3.1.1. Locomotor Activity

**Figure 1:**
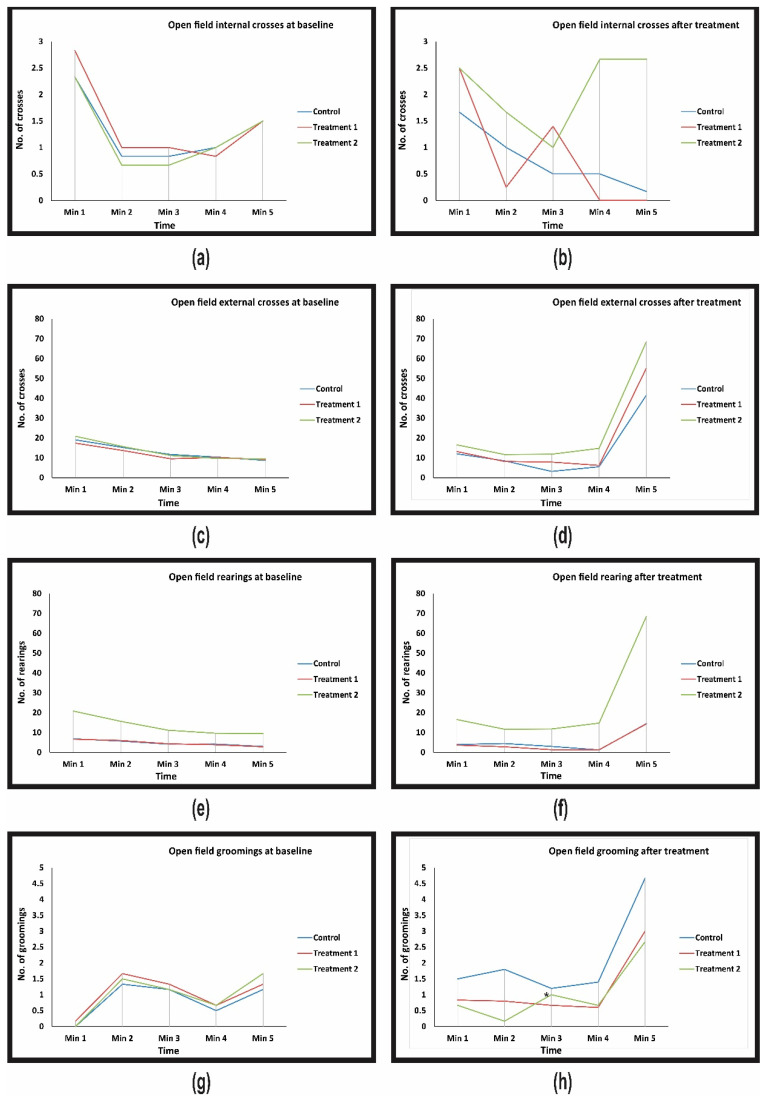
Timely evolution of open-field neurological tests conducted on control and treated rats. (**a**) The number of crossing over squares in internal regions at baseline; (**b**) The number of crossing over squares in internal regions after treatments; (**c**) The number of crossing over squares in external regions at baseline; (**d**) The number of crossing over squares in external regions after treatments; (**e**) Number of rearings at baseline; (**f**) Number of rearings after treatments; (**g**) Number of groomings at baseline; (**h**) Number of groomings after treatment. * Treatment effect is significant compared with the control group.

At the baseline, the locomotor activity parameters analyzed by open field tests were homogenous between the groups (Table 1). After 3 months of treatment, in the control group, the effect of ageing determined a decrease of the locomotor and exploratory activity translated by an increase in the latency to leave from the middle square, a decrease in the number of crossing over internal and external squares and a decrease in rearings (Table 1). The treatment with supplement 1 determined a significant decrease of latency, associated with a decrease in all the other locomotor activity parameters but without reaching the statistical significance compared with the control group (Table 1, Figure 1). In treatment group 2, the effects of supplement 2 were more pronounced in increasing the locomotor activity with a significant decrease of the latency time and a significant increase in rearing, especially at 5 min (Table 1, Figure 1). Compared with supplement 1, supplement 2 was significantly better in increasing the number of rearings (Table 1).

The results of the treatment effects analysis confirmed the beneficial effects of the treatments with supplements 1 and 2 in decreasing the ageing effect on locomotor activity. The treatment with the supplement 2 determined a significant decrease in the latency with 1.42 s followed by a decrease of 1.18 s in rats treated with supplement 1 that was below the values of the control group at the same time point (2.58 s) and also below the values of the controls at baseline (1.50 s) (Table 2).

#### 3.1.2. The Level of Stress

At the baseline, the parameters of the levels of stress analyzed by open field tests were homogenous between the groups (Table 1). After 3 months of treatment, in the control group, the effect of ageing determined a slow increase in the stress level demonstrated by a slow increase in the number of grooming and boluses. The treatment with supplement 2 determined a significant decrease in the number of grooming in the 3rd minute compared with the control group (Table 1 and Figure 1).

The results of the regression of the differences and the treatment effects analysis both identified a significant decrease in the stress level compared with the controls translated by a significant decrease in the number of groomings at 3 min with 0.66 in the group treated with supplement 2 compared with control, followed by a marginal effect of the treatment 1 without reaching the statistical significance. None of the other open field neurological test parameters provided statistically significant results (Table 2 and Table 3).

### 3.2. The Elevated Plus-Maze Test

#### 3.2.1. Exploratory Activity

At the baseline, the exploratory activity parameters analyzed by elevated plus maze tests were homogenous between the groups. After 3 months of treatment, in the control group, we saw that aging’s effect determined a decrease of the exploratory activity translated by a significant decline in the number of bendings, rearings, time spent in open sleeves and time spend in the center (Table 4). The treatment with the supplement 2 determined a significant increase of exploratory activity translated in the increase in the number of bendings and rearings and the time spent in the open sleeves and in the center, followed by the treatment with supplement 2 (Table 4).

The results of the regression analysis of differences to baseline and treatment effects analysis confirmed the beneficial effects of the treatments with supplements 1 and 2 in decreasing the ageing effect on exploratory activity in the elevated plus-maze test. The treatment with supplement 2 determined a significant increase in the number of bendings (with 1.17), the number of rearings (with 3.5), time spent in the open arms (with 10.22) and time spent in the center (with 13.50) compared with control. In the group treated with supplement 1 the effects were significant only for increasing the time spent in the open arms (with 8.35) and the time spent in the center (with 17.27) compared with control (Table 5 and Table 6). 

#### 3.2.2. The Level of Stress

At the baseline, the stress level parameters analyzed by elevated plus maze tests were homogenous between the groups. After 3 months of treatment, in the control group, an increase of the anxiety level was observed, as defined by an increase of the time spent in closed sleeves and of the number of groomings compared with baseline. The treatment with supplement 1 and 2 decreased the anxiety in rats, translated by a significant decrease in the time spent in closed sleeves. In the number of groomings, the effects are more pronounced for supplement 2 (Table 4).

The results of the regression analysis of differences to baseline and treatment effects analysis confirmed the beneficial effects of the treatments with supplements 1 and 2 in decreasing the ageing effect on the level of stress in the elevated plus-maze test. The treatment with supplement 1 determined a significant decrease in the time spend in the dark place with 25.61 s and in the number of groomings with 1.43 compared to the control followed by the treatment with supplement 2 that showed a decrease in the time spent in the dark place with 23.83 s and in the number of groomings with 1.25 compared with the control (Table 5 and Table 6).

### 3.3. The Rotarod Test

**Figure 2:**
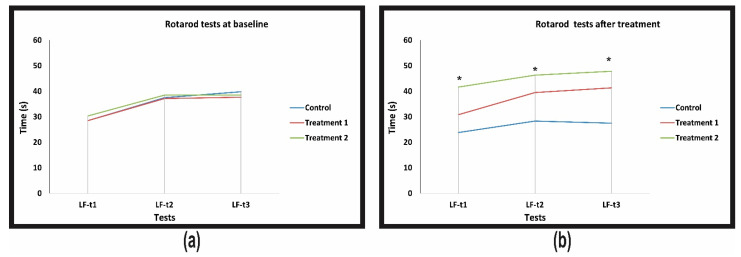
Timely evolution of rotarod neurological tests conducted on control and treated rats. (**a**) Rotarod tests at baseline; (**b**) rotarod tests after treatments. * Statistically significant result *p* < 0.05.

In the rotarod tests, ageing determined a significant decrease in the time spent on the rod in all three consecutive trials (Figure 2). The treatment with supplement 1 annulled the ageing effect, keeping the levels to the same level as the baseline for that group but did not reach statistical significance. The treatment with the supplement 2 annulled the ageing effect and significantly increased the time spent on the rod by the treated rats (Table 7 and Table 8, Figure 2). The regression of differences analysis and the treatment effect analysis supported the effect of treatment with supplement 2 in increasing middle-age rats’ motor performance (Table 7 and Table 8).

## 4. Discussion

In this study, we used a rat model of middle age rats (18 months), which corresponds to 45 years of human age [45] to assess the efficacy of a new dietary supplement containing vitamin C, vitamin D3, zinc and *Centella asiatica* L. extract on locomotor activity and stress level. In rats, similarly to humans, ageing determines a decrease in the exploratory and locomotor activity as well as an increase in the stress level which is supported by our results, with reference to the control group for which ageing after the 3 months treatment which corresponded to 7.5 years of human life [45]. Similar to our findings, Turner et al. on the same strain of Sprang-Dawley 18 months old rats observed that ageing is associated with a significantly less time spent in the open arms in the elevated plus-maze test and a decrease in the time spend on the rod in the rotarod test [46]. Both genetic and environmental factors can influence the locomotor performance of humans. Some studies implicate the long-term-low-dose exposure to xenobiotics and the increase in oxidative stress and inflammation markers [40,41,42] in the stimulation of muscle quality degeneration and neural degeneration, especially in the elderly [4]. In the last years, research interest in dietary supplements with antioxidants and vitamins is increasingly rising in virtue of their potential anti-ageing effects on various levels. In this study, we evaluated for the first time two dose regimens of dietary supplement “Reverse” that contains *Centella asiatica* L. extract, vitamin C (as magnesium ascorbate), zinc (as zinc citrate) and vitamin D3 (as cholecalciferol) on motor performance and stress level of mature rats. Our results suggest that the dose of 2 capsules of Reverse supplement per kg bw of rat (supplement 2) was more effective than 1 capsule of Reverse supplement per kg bw of rat (supplement 1) in improving the motor performance in the rotarod test, in the open field and elevated plus-maze tests and in decreasing the stress level in the open field and elevated plus-maze test as well. The effects were marginal after the treatment with 1 capsule of the Reverse supplement per kg bw of rat (supplement 1) compared to the treatment group 2. The animal model of natural ageing is very similar to the natural human situation, as seen in the examples described above. In this study, we used a middle-age range animal model to simulate all the physiological changes that can appear in the organism with natural ageing that can influence the results of the analysis. The translation of these findings in humans showed that the 2 capsules of Reverse per day regiment are more effective in improving the motor performance and decreasing stress level than 1 capsule of Reverse per day. These findings can be an argument for starting clinical trials with middle-age healthy volunteers for evaluating these effects.

Oxidative stress is implicated in many neurodegenerative diseases [47,48] and is also correlated to the physical decline in the elderly, leading to life quality deterioration. The oxidative stress associated with ageing decreases the muscular strength via muscle mass reduction and impairment of the excitation-contraction coupling, mediated by ryanodine receptors dysfunction associated with oxidation or/and nitrosylation [49]. Studies have demonstrated the age-related increase in protein oxidative damage, oxidative DNA damage and lipid peroxidation in muscles compared to young animals [50]. The consumption of antioxidants can alleviate the physical decline in the elderly and improve life quality [51].

*Centella asiatica* (L.) urban is a plant with proved antioxidant effects. Its properties are associated with the triterpene compounds contained as madecassoside, asiaticoside and asiatic acid and the caffeoylquinic acid derivatives related to the stimulation of the Nrf2-antioxidant response pathway [52]. The ameliorating effects of *Centella asiatica* on cognitive impairment in old animals have been demonstrated in many studies [53,54] but its impact on strengthening motor performance and vigor in healthy elder individuals are scarcely investigated. To the best of our knowledge, locomotor effects were evaluated in a single study, which recruited healthy old human volunteers who received a crude extract of *Centella asiatica,* for 12 weeks. The supplementation showed beneficial effects in improving physical activity by restoring lower extremity muscle strength [55].

Vitamin C is associated with the protection of proteins, lipids, nucleic acids and carbohydrates from oxidative stress. Vitamin C supplements can protect muscles from oxidative damage associated with muscle weakness and decrease physical activity in the elderly [50].

Moreover, studies have shown that zinc supplementation can mitigate the risk of osteoporosis and improve the markers associated with bone formation such as alkaline phosphatase and osteocalcin, recovering mobility, especially in the elderly population [56].

The levels of vitamin D decrease in the elderly mainly due to the reduction of intestinal absorption, of vitamin D synthesis and of vitamin D renal activation in addition to the reduction in calcium absorption [22]. Vitamin D deficiency in the elderly is primarily associated with the risk of osteoporosis and decreased physical activity along with other non-bone disorders [22].

In our study, the administration of a novel nutraceutical formulation containing a combination of *Centella asiatica* L. extract, vitamin C, zinc and vitamin D3 (as cholecalciferol) acts additively in increased the motor performance in middle age rats. The second dose-regimen was generally more effective than the first one. The supplement effect is associated with multiple mechanisms that decrease oxidative stress damage on muscle and improve new bone formation. More than that, the supplement’s administration also abated the level of stress in animals translated in the decrease of the number of groomings and boluses both in the open field test and elevated plus-maze test with the second dose regiment generally more effective than the first one. Interestingly, for only one parameter of stress level and only one parameter of exploratory activity, the first dose-regimen was more effective compared with the second. This can be related to combined effects of the constituents of the supplement as has been seen in other studies [43,44] and that’s why these findings should also be checked in the human clinical trials.

Although it is important not to extrapolate our findings, the formulation constituents’ antioxidant properties might be responsible for this beneficial effect. A meta-analysis investigating the effects of *Centella asiatica* supplementation on cognition showed that it can improve mood by lowering the anger score and boosting the alert level [57]. Due to high antioxidant effects, vitamin C supplementation decreases the level of anxiety and stress by modulation of oxidative stress increased in these pathologies [58]. Zinc is a trace element implicated in brain and body response to stress. A decreased level of zinc is associated with depression. Studies on rats exposed to repeated psychological stress showed that the stress induces depletion of zinc in the hippocampus and increased cortisol concentration accompanied by a decrease in serum zinc level and an increase in liver zinc level, showing that chronic stress can decrease zinc absorption and increase zinc liver accumulation [59]. A recent meta-analysis presents preclinical and clinical evidence supporting the beneficial role of vitamin D supplementation in anxiety disorders and depressive disorders [60]. Nutrients are known to act synergistically and it has been previously suggested that supplementation with the proper nutrient combination can have an additive effect on health [61,62]. To this direction, Reverse combines nutrients with proven roles in muscle integrity, mobility and stress, as discussed above. However, future studies need to test side-by-side the efficacy of single nutrients to the formulation to investigate the synergy effect. Also, the present study did not investigate the mode of action of this supplement. The supplement contains constituents with known antioxidant features, thus it could be suggested that the beneficial effect of the supplement is by reducing oxidative stress and further experiments will validate this hypothesis. Despite these limitations, the present study stands as a proof-of-concept for using a novel nutraceutical formulation to enhance physical strength and improve life quality.

## 5. Conclusions

In conclusion, we showed that the supplementation with Reverse (Natural Doctor S.A), a novel nutraceutical formulation, can improve the locomotor activity and decrease the stress level in middle-age rats, with higher effects in the second dose regiment. These effects may be associated with the additive beneficial effects of combining *Centella asiatica* L. extract, vitamin C, zinc and vitamin D3 on antioxidant and muscle and bone integrity mechanisms. However, validation experiments are required since each constituent acts through different mechanisms to improve locomotor activity and decrease the stress level. Further studies should evaluate these effects also in humans to demonstrate the potential efficacy in clinical practice.

## Figures and Tables

**Table 1:** Open-field tests result expressed as mean ± standard deviation.

Parameter	Baseline			After Treatment		
	Control	Treatment 1	Treatment 2	Control	Treatment 1	Treatment 2
**Locomotor Activity**						
Latency to leave from the middle square	1.50 ± 1.87	1.00 ± 0.63	1.17 ± 0.75	3.67 ± 1.63	1.83 ± 0.75 *	1.17 ± 0.75 *
Number of crossing over internal squares in min 1	2.33 ± 1.51	2.83 ± 2.14	2.33 ± 1.51	1.67 ± 1.21	2.50 ± 1.05	2.50 ± 1.38
Number of crossing over internal squares in min 2	0.83 ± 1.17	1.00 ± 1.67	0.67 ± 0.82	1.00 ± 0.89	0.25 ± 0.50	1.67 ± 0.58
Number of crossing over internal squares in min 3	0.83 ± 1.33	1.00 ± 2.00	0.67 ± 1.21	0.50 ± 0.84	1.40 ± 1.52	1.00 ± 0.00
Number of crossing over internal squares in min 4	1.00 ± 1.26	0.83 ± 2.04	1.00 ± 1.26	0.50 ± 1.22	0.00 ± 0.00	2.67 ± 1.15
Number of crossing over internal squares in min 5	1.50 ± 1.76	1.50 ± 2.35	1.50 ± 2.35	0.17 ± 0.41	0.00 ± 0.00	2.67 ± 1.53
Number of crossing over internal squares in all 5 min	6.67 ± 5.09	7.17 ± 8.80	6.00 ± 5.22	4.17 ± 4.58	3.83 ± 2.48	6.67 ± 5.50
Number of crossing over external squares in min 1	19.00 ± 10.14	17.33 ± 9.22	20.83 ± 10.50	14.33 ± 8.94	20.83 ± 8.77	20.83 ± 7.52
Number of crossing over external squares in min 2	15.17 ± 10.15	13.67 ± 6.31	15.67 ± 9.42	12.00 ± 6.04	13.17 ± 9.35	16.50 ± 10.67
Number of crossing over external squares in min 3	11.67 ± 9.14	9.50 ± 5.01	11.17 ± 8.93	8.50 ± 5.43	8.17 ± 5.04	11.67 ± 8.19
Number of crossing over external squares in min 4	10.33 ± 9.85	10.17 ± 3.43	9.67 ± 8.91	3.17 ± 2.48	7.83 ± 7.88	11.80 ± 8.58
Number of crossing over external squares in min 5	8.67 ± 10.58	9.17 ± 4.45	9.50 ± 8.98	5.50 ± 6.53	6.20 ± 6.18	14.75 ± 7.80
Number of crossing over external squares in all 5 min	64.83 ± 43.93	66.83 ± 31.43	66.83 ± 43.52	41.50 ± 21.47	55.17 ± 30.73	68.50 ± 43.50
Number of rearings in min 1	6.83 ± 3.54	6.67 ± 1.51	7.00 ± 1.79	5.80 ± 1.92	5.17 ± 2.04	7.33 ± 3.39
Number of rearings in min 2	5.67 ± 3.44	6.00 ± 2.10	5.33 ± 0.52	4.00 ± 2.55	3.67 ± 2.94	5.83 ± 2.14
Number of rearings in min 3	4.17 ± 3.06	4.33 ± 1.97	4.33 ± 2.34	4.50 ± 1.73	2.83 ± 2.32	5.80 ± 0.84
Number of rearings in min 4	4.20 ± 3.35	3.83 ± 2.48	3.17 ± 2.64	3.00 ± 1.87	1.33 ± 1.75	4.25 ± 0.96
Number of rearings in min 5	3.00 ± 3.08	2.83 ± 1.17	1.67 ± 0.82	1.25 ± 1.50	1.33 ± 2.16	1.67 ± 1.37 *^,$^
Number of rearings in all 5 min	22.67 ± 13.88	23.67 ± 4.55	21.50 ± 5.50	14.50 ± 9.93	14.33 ± 8.89	22.83 ± 7.05
**The Level of Stress**						
Number of groomings in min 1	0.00 ± 0.00	0.17 ± 0.41	0.00 ± 0.00	0.00 ± 0.00	0.40 ± 0.55	1.00 ± 0.00
Number of groomings in min 2	1.33 ± 1.21	1.67 ± 1.21	1.50 ± 0.84	1.50 ± 1.29	0.83 ± 0.75	0.67 ± 0.82
Number of groomings in min 3	1.17 ± 0.75	1.33 ± 1.03	1.17 ± 0.75	1.80 ± 0.84	0.80 ± 1.10	0.17 ± 0.41 *
Number of groomings in min 4	0.50 ± 0.55	0.67 ± 0.52	0.67 ± 0.52	1.20 ± 0.45	0.67 ± 0.52	1.00 ± 0.71
Number of groomings in min 5	1.17 ± 1.33	1.33 ± 1.03	1.67 ± 0.82	1.40 ± 1.14	0.60 ± 0.89	0.67 ± 0.52
Number of groomings in all 5 min	4.17 ± 3.19	5.17 ± 2.79	5.00 ± 2.00	4.67 ± 3.72	3.00 ± 2.28	2.67 ± 1.51
Number of boluses	2.33 ± 2.34	2.17 ± 1.33	1.83 ± 1.17	2.83 ± 1.94	2.17 ± 0.98	2.00 ± 1.10

* Post-hoc test, comparison with the control group after the treatment, *p* < 0.05. ^$^ Post-hoc test, comparison with treatment 1 group after the treatment, *p* < 0.05.

**Table 2:** Regression analysis of each treatment group compared with the control group.

Parameter	Treatment	B Coefficient	[95% Confidence Interval]	*p*
**Locomotor Activity**				
Latency to leave from the middle square	Treatment 1	−0.50 ± 0.81	[−2.22 ± 1.22]	0.544
	Treatment 2	−0.33 ± 0.82	[−2.09 ± 1.42]	0.691
Number of crossing over internal squares in min 1	Treatment 1	0.45 ± 0.47	[−0.85 ± −0.31]	0.085
	Treatment 2	0.32 ± 0.45	[−0.70 ± 2.10]	0.217
Number of crossing over internal squares in min 2	Treatment 1	0.33 ± 1.51	[−2.89 ± 3.56]	0.829
	Treatment 2	0.83 ± 1.04	[−1.37 ± 3.04]	0.434
Number of crossing over internal squares in min 3	Treatment 1	−1.00 ± 0.89	[−2.89 ± 0.89]	0.278
	Treatment 2	0.00 ± 0.51	[−1.08 ± 1.08]	1.000
Number of crossing over internal squares in min 4	Treatment 1	0.50 ± 1.16	[−1.97 ± 2.97]	0.672
	Treatment 2	0.33 ± 0.61	[−0.98 ± 1.64]	0.596
Number of crossing over internal squares in min 5	Treatment 1	−0.33 ± 0.90	[−2.25 ± 1.59]	0.716
	Treatment 2	0.83 ± 0.40	[−0.10 ± 1.69]	0.057
Number of crossing over internal squares in all 5 min	Treatment 1	−0.17 ± 1.17	[−2.65 ± 2.32]	0.888
	Treatment 2	1.17 ± 0.78	[−0.49 ± 2.83]	0.155
Number of crossing over external squares in min 1	Treatment 1	−0.83 ± 4.40	[−10.21 ± 8.55]	0.852
	Treatment 2	1.17 ± 0.47	[−0.85 ± 3.30]	0.125
Number of crossing over external squares in min 2	Treatment 1	8.17 ± 3.31	[1.11 ± 15.22]	0.226
	Treatment 2	4.67 ± 2.59	[−0.86 ± 10.19]	0.092
Number of crossing over external squares in min 3	Treatment 1	4.67 ± 3.89	[−3.63 ± 12.96]	0.249
	Treatment 2	6.00 ± 2.66	[−2.34 ± 7.66]	0.178
Number of crossing over external squares in min 4	Treatment 1	1.83 ± 4.01	[−6.71 ± 10.38]	0.654
	Treatment 2	3.67 ± 3.40	[−3.58 ± 10.91]	0.298
Number of crossing over external squares in min 5	Treatment 1	4.83 ± 4.52	[−4.81 ± 14.48]	0.302
	Treatment 2	7.33 ± 3.55	[−1.23 ± 14.90]	0.060
Number of crossing over external squares in all 5 min	Treatment 1	−0.83 ± 4.47	[−10.36 ± 8.69]	0.855
	Treatment 2	3.50 ± 3.24	[−3.40 ± 10.40]	0.297
Number of rearings in min 1	Treatment 1	11.67 ± 13.85	[−17.86 ± 41.19]	0.413
	Treatment 2	25.00 ± 11.57	[−3.34 ± 29.66]	0.067
Number of rearings in min 2	Treatment 1	0.50 ± 1.28	[−2.24 ± 3.24]	0.703
	Treatment 2	2.33 ± 1.36	[−0.56 ± 5.23]	0.106
Number of rearings in min 3	Treatment 1	0.00 ± 2.21	[−4.71 ± 4.71]	1.000
	Treatment 2	2.83 ± 1.78	[−0.96 ± 6.62]	0.132
Number of rearings in min 4	Treatment 1	−0.33 ± 1.27	[−3.04 ± 2.37]	0.796
	Treatment 2	1.67 ± 0.78	[0.00 ± 3.33]	0.058
Number of rearings in min 5	Treatment 1	−1.50 ± 1.69	[−5.10 ± 2.10]	0.388
	Treatment 2	0.67 ± 0.95	[−1.37 ± 2.70]	0.496
Number of rearings in all 5 min	Treatment 1	0.17 ± 1.62	[−3.29 ± 3.62]	0.919
	Treatment 2	1.67 ± 1.36	[−1.23 ± 4.56]	0.239
**The Level of Stress**				
Number of groomings in min 1	Treatment 1	9.50 ± 4.40	[0.13 ± 18.87]	0.047 *
	Treatment 2	−1.17 ± 5.84	[−13.61 ± 11.27]	0.844
Number of groomings in min 2	Treatment 1	0.17 ± 0.17	[−0.19 ± 0.52]	0.333
	Treatment 2	0.33 ± 0.21	[−0.12 ± 0.78]	0.135
Number of groomings in min 3	Treatment 1	−0.52 ± 0.28	[−2.23 ± −0.14]	0.062
	Treatment 2	−0.63 ± 0.20	[−1.74 ± −0.24]	0.040 *
Number of groomings in min 4	Treatment 1	−0.25 ± 0.37	[−2.00 ± 3.00]	0.261
	Treatment 2	−1.33 ± 0.49	[−2.39 ± 0.28]	0.117
Number of groomings in min 5	Treatment 1	−0.50 ± 0.34	[−1.23 ± 0.23]	0.164
	Treatment 2	−0.33 ± 0.38	[−1.14 ± 0.48]	0.394
Number of groomings in all 5 min	Treatment 1	−0.83 ± 0.65	[−2.23 ± 0.56]	0.222
	Treatment 2	−1.00 ± 0.52	[−2.10 ± 0.10]	0.072
Number of boluses	Treatment 1	−1.67 ± 0.97	[−4.74 ± −2.60]	0.310

* Statistically significant result, *p* < 0.05.

**Table 3:** Averaged treatment effects for open field tests.

Parameter	Treatment 1 vs. Control Coefficient ATET [Confidence Interval] (*p*)	Treatment 2 vs. Control Coefficient ATET [Confidence Interval] (*p*)	Potential Outcome Mean
**Locomotor activity**			
Latency to leave from the middle square	−1.18 [−2.38–0.18] (0.05) *	−1.41 [−2.58–−0.26] (0.017) *	2.58
Number of crossing over internal squares in min 1	0.70 [−0.57–1.97] (0.280)	0.42 [−0.63–1.46] (0.434)	2.00
Number of crossing over internal squares in min 2	−0.32 [−1.28–0.64] (0.518)	−1.67 [−0.88–0.55] (0,001)	0.92
Number of crossing over internal squares in min 3	−0.53 [−0.68–1.75] (0.389)	1.63 × 10^−17^ [−0.75–0.75] (1.000)	0.67
Number of crossing over internal squares in min 4	−0.25 [−1.39–0.89] (0.667)	0.42 [−0.59–1.42] (0.417)	0.75
Number of crossing over internal squares in min 5	0.67 [−1.29–1.42] (0.923)	0.58 [−0.73–1.90] (0.384)	0.83
Number of crossing over internal squares in all 5 min	0.48 [−4.37–5.33] (0.845)	0.92 [−2.88–4.72] (0.636)	5.42
Number of crossing over external squares in min 1	2.73 [−4.90–10.36] (0.483)	4.17 [−2.78–11.12] (0.240)	16.67
Number of crossing over external squares in min 2	0.42 [−6.42–7.26] (0.905)	3.5 [−3.57–10.57] (0.332)	12.58
Number of crossing over external squares in min 3	−0.78 [−5.79–4.22] (0.759)	1.33 [−4.62–7.29] (0.661)	10.08
Number of crossing over external squares in min 4	0.85 [−4.45–6.15] (0.753)	3.00 [−3.28–9.28] (0.349)	6.75
Number of crossing over external squares in min 5	0.52 [−5.28–6.32] (0.861)	2.58 [−4.11–9.28] (0.449)	7.08
Number of crossing over external squares in all 5 min	7.93 [−19.41–35.28] (0.570)	14.50 [−14.96–43.58] (0.335)	53.17
Number of rearings in min 1	−2.33 [−2.32–1.86] (0.827)	1.33 [−0.93–3.59] (0.247)	5.83
Number of rearings in min 2	0.10 [−2.35–2.55] (0.936)	1.08 [−0.85–3.01] (0.272)	4.50
Number of rearings in min 3	0.22 [−1.84–2.27] (0.836)	1.00 [−0.97–2.97] (0.320)	3.58
Number of rearings in min 4	−0.10 [−2.22–2.02] (0.926)	1.20 × 10^−16^ [−1.97–1.97] (1.000)	3.00
Number of rearings in min 5	0.73 [−0.95–2.42] (0.394)	1.30 × 10^−16^ [−1.42–1.42] (1.000)	1.67
Number of rearings in all 5 min	0.72 [−7.83–9.26] (0.869)	3.58 [−3.83–11.00] (0.344)	18.58
**The Level of Stress**			
Number of groomings in min 1	0.30 [0.02–0.58] (0.038) *	0.17 [−0.04–0.38] (0.121)	8.84 × 10^−33^
Number of groomings in min 2	0.13 [−0.81–1.07] (0.781)	−0.08 [−0.89–0.73] (0.840)	1.17
Number of groomings in min 3	−0.63 [−1.32–0.05] (0.069)	−0.66 [−1.31–−0.03] (0.041) *	1.33
Number of groomings in min 4	−0.05 [−0.49–0.39] (0.824)	3.51 × 10^−17^ [−0.48–0.48] (1.000)	0.75
Number of groomings in min 5	−0.47 [−1.27–0.34] (0.257)	3.02 × 10^−17^ [−0.79–0.79] (1.000)	1.17
Number of groomings in all 5 min	−0.72 [−3.08–1.65] (0.553)	−0.58 [−2.70–1.53] (0.590)	4.42
Number of boluses	−0.58 [−1.86–0.69] (0.371)	−0.67 [−1.93–0.60] (0.301)	2.58

* Statistically significant result, *p* < 0.05.

**Table 4:** Elevated plus-maze tests result expressed as mean ± standard deviation.

Parameter	Baseline			After Treatment		
	Control	Treatment 1	Treatment 2	Control	Treatment 1	Treatment 2
**Exploratory activity**						
Number of bendings	1.50 ± 1.05	1.33 ± 1.03	1.50 ± 0.55	1.00 ± 0.82	1.47 ± 1.07 *	3.67 ± 1.21 *
Number of rearings	5.50 ± 1.87	5.00 ± 1.41	5.33 ± 1.03	3.50 ± 0.58	4.89 ± 2.60	8.22 ± 1.91 *
Time spent in the open arms (seconds)	19.50 ± 5.32	18.17 ± 6.62	18.17 ± 4.07	16.25 ± 5.56	19.17 ± 10.53 *	32.31 ± 8.30 *
Time spent in the center (seconds)	44.50 ± 6.19	49.67 ± 11.76	46.50 ± 11.67	31.25 ± 5.50	46.86 ± 15.47 *	57.06 ± 7.94 *
**The Level of Stress**						
Time spend in the dark place	236.00 ± 5.76	232.17 ± 16.10	235.33 ± 12.09	252.50 ± 7.14	233.97 ± 24.44 *	210.64 ± 11.70 *
Number of groomings	1.83 ± 1.17	1.50 ± 1.05	1.67 ± 0.82	3.00 ± 1.41	1.64 ± 1.48 *	0.22 ± 0.40 *

* Post-hoc test, comparison with the control group after the treatment, *p* < 0.05.

**Table 5:** Regression analysis of each treatment group compared with the control group.

Parameter	Treatment	B Coefficient	[95% Confidence Interval]	*p*
**Exploratory Activity**				
Number of bendings	Treatment 1	0.85 ± 0.22	[−0.18 ± 2.68]	0.065
	Treatment 2	1.33 ± 1.12	[0.92 ± 2.26]	0.006 *
Number of rearings	Treatment 1	1.43 ± 0.49	[0.12 ± 3.17]	0.062
	Treatment 2	2.33 ± 0.47	[1.33 ± 3.34]	0.000 **
Time spent in the open arms (seconds)	Treatment 1	6.17 ± 0.65	[0.76 ± −10.77]	0.005 *
	Treatment 2	9.27 ± 0.54	[3.32 ± 19.01]	0.001 *
Time spent in the center (seconds)	Treatment 1	16.17 ± 2.77	[10.25 ± 22.08]	<0.001 **
	Treatment 2	13.33 ± 3.07	[9.79 ± 21.87]	<0.001 **
**The Level of Stress**				
Time spent in the dark place	Treatment 1	−28.00 ± 4.53	[−11.35 ± −33.65]	<0.001 **
	Treatment 2	−23.00 ± 4.71	[−12.97 ± −33.03]	<0.001 **
Number of groomings	Treatment 1	−0.52 ± 0.25	[−2.36 ± −00.25]	<0.001 **
	Treatment 2	−0.83 ± 1.34	[−1.48 ± 00.28]	<0.001 **

* Statistically significant result *p* < 0.05; ** Statistically significant result *p* < 0.001.

**Table 6:** Averaged treatment effects for the elevated plus-maze test.

Parameter	Treatment 1 vs. Control Coefficient ATET [Confidence Interval] (*p*)	Treatment 2 vs. Control Coefficient ATET [Confidence Interval] (*p*)	Potential Outcome Mean
**Exploratory activity**			
Number of bendings	0.93 [−0.02–1.89] (0.055)	1.17 [0.33–2.00] (0.006) *	1.17
Number of rearings	1.48 [−0.17–3.14] (0.079)	3.50 [1.27–5.72] (0.002) *	4.42
Time spent in the open arm (seconds)	8.35 [2.43–14.27] (0.006) *	10.33 [3.54–17.12] (0.003) *	16.75
Time spent in the center (seconds)	17.27 [8.78–25.75] (<0.001) *	13.50 [5.83–21.17] (0.001) *	38.83
**The Level of Stress**			
Time spent in the dark place	−25.61 [−37.95–−13.28] (<0.001) *	−23.83 [−35.41–−12.25] (<0.001) *	244.42
Number of groomings	−1.43 [−2.38–−0.49] (0.003) *	−1.25 [−2.13–−0.36] (0.006) *	2.33

* Statistically significant result, *p* < 0.05.

**Table 7:** Regression analysis of each treatment group compared with the control group.

Parameter	Treatment	B Coefficient	[95% Confidence Interval]	*p*
Latency to fall—t1	Treatment 1	3.17 ± 5.22	[4.28 ± −8.08]	0.424
	Treatment 2	4.33 ± 1.77	[1.72 ± −4.94]	0.000 **
Latency to fall—t2	Treatment 1	−0.32 ± 1.66	[−1.46 ± 1.20]	0.702
	Treatment 2	11.01 ± 2.29	[8.15 ± 13.89]	0.000 **
Latency to fall—t3	Treatment 1	−2.21 ± 3.60	[−6.33 ± 4.17]	0.653
	Treatment 2	12.00 ± 3.88	[8.73 ± 17.27]	0.011 *

* Statistically significant result *p* < 0.05; ** Statistically significant result *p* < 0.001.

**Table 8:** Averaged treatment effects for rotarod tests.

Parameter	Treatment 1 vs. Control Coefficient ATET [Confidence Interval] (*p*)	Treatment 2 vs. Control Coefficient ATET [Confidence Interval] (*p*)	Potential Outcome Mean
Latency to fall–t1	1.73 [−2.21–5.68] (0.389)	9.83 [4.73–14.94] (<0.001) *	26.17
Latency to fall–t2	−0.42 [−6.30–5.47] (0.890)	9.50 [1.86–17.14] (0.015) *	32.92
Latency to fall–t3	−1.17 [−9.24–6.91] (0.777)	9.50 [0.07–18.92] (0.048) *	33.67

* Statistically significant result, *p* < 0.05.

## Data Availability

The dataset presented in this study is available from the corresponding author upon reasonable request.
